# Characterization of the complete chloroplast genome sequence of submerged macrophyte *Stuckenia pectinata* (Potamogetonaceae) and its phylogenetic position

**DOI:** 10.1080/23802359.2019.1703595

**Published:** 2019-12-18

**Authors:** Jian-Juan Tian, Xue Zhang, Zhen-Dian Liu, Shi-Kang Shen

**Affiliations:** a School of Life Sciences, Yunnan University, Kunming, China;; b Breeding Base for State Key Laboratory of Land Degradation and Ecological Restoration in Northwest China, Key Laboratory for Restoration and Reconstruction of Degraded Ecosystem in Northwest China of Ministry of Education, Ningxia University, Yinchuan, China;; c School of Ecology and Environmental Sciences and Yunnan Key Laboratory for Plateau Mountain Ecology and Restoration of Degraded Environments, Yunnan University, Kunming, China

**Keywords:** *Stuckenia pectinata*, Plateau lake, adaptability, phylogenetic, chloroplast genome

## Abstract

*Stuckenia pectinata* is widely distributed submerged macrophyte in the world. Herein, the complete chloroplast genome of this species was assembled and characterized using whole genome next-generation sequencing. The complete chloroplast genome showed a circular genome of 156,669 bp size with 36.5% GC content. The genome is of typical structure and contain a pair of inverted repeat (IR) regions with 26,074 bp, separated by one large single-copy (LSC) with 86,285 bp, and one small single-copy (SSC) regions with 18,236 bp. *De novo* assembly and annotation showed the presence of 131 unique genes with 85 protein-coding genes, 38 tRNA genes, and eight rRNA genes. A maximum-likelihood phylogenomic tree reconstructed based on 15 chloroplast genomes reveals that *S. pectinata* is most closely related to *Zostera marina.*

*Stuckenia* is a genus of Potamogetonaceae identified by the characteristics of long leaf sheaths, characteristic leaf and peduncle anatomy (Kaplan 2008). *Stuckenia pectinata* is the most widespread species of *Stuckenia* and occurs in all continents of the world (Du and Wang 2016). The species play an important role in nutrients and heavy metals removal in the heavy polluted water (Malkiat et al. 2018). However, *Stuckenia* species always show a wide range of morphological variation in the field (Kaplan 2008). A comprehensive complete chloroplast genome information would provide useful DNA barcodes for future species identification and phylogeny. Herein, we first assembled and characterized the complete chloroplast genome for *S. pectinata* using next-generation sequencing technology. Such information will pave the way for future studies on phylogenetic of *S. pectinata*.

The *S. pectinata* plant used for genome sequencing was collected from the lake of Dianchi in Yunnan Province, China (E: 102°46′24.86″, N: 24°49′24.89″). The specimen is stored at Yunnan University Herbarium-Laboratory of plant germplasm conservation and systematic adaptation (HYN-SSK190015). Total genomic DNA was extracted using a modified cetyltrimethylammonium bromide (CTAB) method (Doyle 1987). The sequencing library was constructed and quantified, and then the paired-end (PE) libraries were generated using Illumina HiSeq 2500 platform. The whole genome sequencing was conducted by Softgene (Beijing, China). We assembled the short reads into contigs using SPAdes, connected all contigs with Bandage, and manually removed redundant contigs. We mapped reads to the genome to check, proofread, and patch and finally obtained cycle complete plastomes. The cp genome was annotated through DOGMA (Wyman et al. 2004), and the boundaries of start and stop codons, and intron/exon were checked manually using Geneious version 8.1.4. We confirmed all tRNA genes using online tRNAscan-SE (Schattner et al. 2005). The final complete plastomes were deposited in GenBank with accession numbers MN661144.

The cp genome of *S. pectinata* is a circular molecule of 156,669 base pairs (bp), with a pair of Inverted Repeats (IR) of 26,074 bp, separated by a large (LSC, 86,285 bp) and a small single copy (SSC, 18,236 bp). The overall GC content of *S. pectinata* cp genome is 36.5% and the corresponding values in IR, LSC and SSC regions are 42.7%, 34.2%, and 29.4%, respectively. The cp genomes were annotated with 131 genes, including 85 protein-coding genes, 38 tRNA genes, and eight rRNA genes. A total of 89 simple sequence repeats (SSRs) were detected using the online software MISA (http://pgrc.ipk-gatersleben.de/misa/，Beier et al., 2017). The numbers of mono-, di-, tri- and tetra- nucleotides SSRs are 49, 21, 4 and 15, respectively.

To obtain insight into the position of *S. pectinata*, we performed a maximum-likelihood phylogenomic analysis using the chloroplast genomes sequences of 15 species (*Thalassia hemprichii* as outgroup) in PAUP version 4.0a with 1000 bootstrap replicates (Swofford 2002). The phylogenetic tree indicated that *S. pectinata* has closer relationship with *Zostera marina* than other species with 100% bootstrap value (Figure 1). The complete chloroplast genome provides valuable genomic resources of *S. pectinata* that can be used to reconstruct its phylogeny, evaluate genetic variations, and develop utilization strategy.

**Figure 1.**
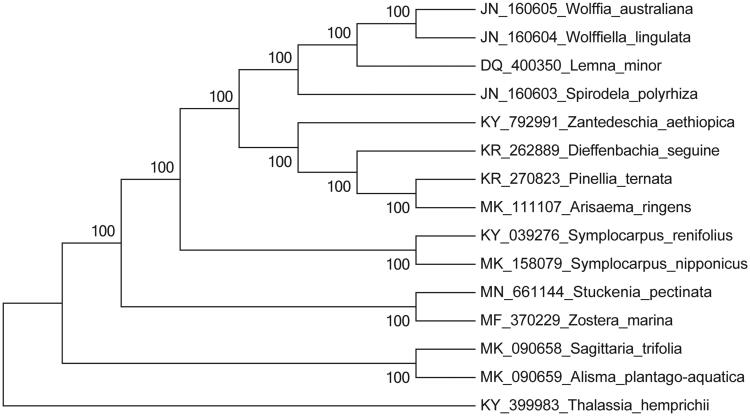
Phylogenetic position of *Stuckenia pectinata* based on the complete chloroplast genome sequences of 15 species. Bootstraps were shown next to the node.
